# The DOF-Domain Transcription Factor ZmDOF36 Positively Regulates Starch Synthesis in Transgenic Maize

**DOI:** 10.3389/fpls.2019.00465

**Published:** 2019-04-12

**Authors:** Jiandong Wu, Long Chen, Mingchao Chen, Wei Zhou, Qing Dong, Haiyang Jiang, Beijiu Cheng

**Affiliations:** National Engineering Laboratory of Crop Stress Resistance, College of Life Science, Anhui Agricultural University, Hefei, China

**Keywords:** *Zea mays* L., *ZmDOF36*, starch synthesis, transcriptional regulation, endosperm

## Abstract

Starch synthesis is a complex process that influences crop yield and grain quality in maize. Many key enzymes have been identified in starch biosynthesis; however, the regulatory mechanisms have not been fully elucidated. In this study, we identified a DOF family gene, *ZmDOF36*, through transcriptome sequencing analysis. Real-time PCR indicated that *ZmDOF36* was highly expressed in maize endosperm, with lower expression in leaves and tassels. *ZmDOF36* is a typical DOF transcription factor (TF) that is localized to the nucleus and possesses transcriptional activation activity, and its transactivation domain is located in the C-terminus (amino acids 227–351). Overexpression of *ZmDOF36* can increase starch content and decrease the contents of soluble sugars and reducing sugars. In addition, abnormal starch structure in transgenic maize was also observed by scanning electron microscopy (SEM). Furthermore, the expression levels of starch synthesis-related genes were up-regulated in *ZmDOF36*-expressing transgenic maize. ZmDOF36 was also shown to bind directly to the promoters of six starch biosynthesis genes, *ZmAGPS1a*, *ZmAGPL1*, *ZmGBSSI*, *ZmSSIIa*, *ZmISA1*, and *ZmISA3* in yeast one-hybrid assays. Transient expression assays showed that ZmDOF36 can activate the expression of *ZmGBSSI* and *ZmISA1* in tobacco leaves. Collectively, the results presented here suggest that ZmDOF36 acts as an important regulatory factor in starch synthesis, and could be helpful in devising strategies for modulating starch production in maize endosperm.

## Introduction

Maize (*Zea mays* L.) is one of the most important food and feed crops in the world and has enormous economic and biological significance (Wu and Messing, 2014). With the completion of the maize genome sequence, it has also become an important model organism for biological studies in addition to being a resource for food, fuel, and feed. The maize endosperm contains ∼10% protein and ∼70% starch (Nelson and Pan, 1995). Starch is pivotal for maize yield and quality, and is the major component of the dry seed weight (Flint-Garcia et al., 2009).

Starch generally consists of two major fractions, linear amylose, and branched amylopectin (Morell and Myers, 2005; Tetlow, 2006). In general, amylopectin comprises about 75% of the starch, whereas amylose accounts for 25%. Amylose is an unbranched linear polymer composed of α-1,4 bonds (fewer than 0.5% a-1,6 linkages), while amylopectin is a highly branched molecule with α-1,4 bonds and 5% α-1,6 linkages (Bresolin et al., 2006; Perez and Bertoft, 2010). The ratio of amylose/amylopectin is crucial for the appearance, structure, and quality of the starch for food products and processing (Hong and Huber, 2015). Amylose is primarily responsible for the starch gel qualities. Amylopectin is typically thought to affect the crystalline granules and paste thickening (Whitt et al., 2002). In the past several decades, the starch metabolic pathways have been well characterized in a number of studies on the key enzymes involved in starch synthesis.

Starch biosynthesis in the cereal endosperm requires the coordinated activities of several different classes of enzymes that include ADP glucose pyrophosphorylase (AGPase), granule-bound starch synthase (GBSS), soluble starch synthase (SS), starch branching enzyme (BE), and starch debranching enzyme (DBE) (Whitt et al., 2002; Jeon et al., 2010). AGPase is a key rate-limiting enzyme that catalyzes the first step in the starch biosynthesis pathway. AGPase consists of two subunits that are encoded by the *Shrunken 2* (*Sh2*) and *Brittle 2* (*Bt2*) genes (Saripalli and Gupta, 2015). In maize, the AGPase gene family consists of seven genes (*ZmAGPS1a*, *ZmAGPS1b*, *ZmAGPS2*, *ZmAGPL1*, *ZmAGPL2*, Z*mAGPL3*, and *ZmAGPL4*). GBSS, encoded by the *WAXY* gene, is primarily responsible for amylose synthesis (Tsai, 1974), whereas amylopectin biosynthesis is mainly catalyzed by SS, BE, and DBE (Myers et al., 2000). In recent years, an increasing number of studies have also shown that a few starch biosynthesis enzymes can regulate starch structure and content by forming stable complexes (Tetlow et al., 2004; Fujita et al., 2006; Hennen-Bierwagen et al., 2008). In maize, for example, ISA2 and SSIII work in a coordinated fashion (Lin et al., 2012).

In addition to the key enzymes, many studies have shown that transcription factors (TFs) are able to regulate starch synthesis by binding to the promoters of starch biosynthesis genes. *SUSIBA2*, one member of the WRKY TF family, is involved in starch synthesis through an interaction with the *iso1* promoter (Sun et al., 2003). *RSR1* (Rice Starch Regulator 1), an AF2/EREBP-type TF, plays a negative regulatory role in starch synthesis (Fu and Xue, 2010). *MADS29* was also demonstrated to regulate starch synthesis and influence seed development (Yin and Xue, 2012). *OsbZIP58* was reported to directly regulate the expression of starch synthesis genes in rice, and mutations in this gene decrease the total starch and amylose contents (Wang et al., 2013). In maize, a number of TF genes that are highly expressed in the endosperm were identified through transcriptome sequencing (Li et al., 2014). Among these TFs, a few had been previously demonstrated to function as key regulatory factors in starch biosynthesis in recent studies. *ZmbZIP91* can induce the expression of starch synthesis genes in the endosperm, and can rescue the mutant phenotype in the *Arabidopsis* *vip1* mutant (*AtVIP1* is homologous to *ZmbZIP91*), resulting in normal starch synthesis (Chen et al., 2016). *ZmZHOUPI*, a bHLH-type TF, plays important roles in the starch synthesis and seed development (Grimault et al., 2015; Feng et al., 2018). Although several TFs involved in starch synthesis have been identified and studied, the mechanisms underlying the regulatory network that controls starch biosynthesis are not well characterized. Therefore, identification of central factors involved in starch synthesis will be important for a complete understanding of the regulation of starch synthesis.

DOF proteins are a family of plant-specific TFs that contain a conserved DNA binding Dof domain that recognizes a *cis-*regulatory element with the common core sequence AAAG (Yanagisawa, 2002). During the process of maize seed development, DOF1 has been reported to up-regulate the expression of gamma-zein (Marzabal et al., 2008). Additionally, the prolamin-box binding factor (PBF) Dof protein associated with seed development has been reported in barley, wheat, and finger millet (Mena et al., 2002; Dong et al., 2007).

In this study, we present the identification of *ZmDOF36* as a candidate regulator of starch synthesis based on previous transcriptome data (Sekhon et al., 2013; Li et al., 2014). Although high expression levels of *ZmDOF36* have been demonstrated in the maize endosperm (Li et al., 2014), the exact function of this gene is still not well characterized. Here, we analyzed the expression patterns and functional properties of *ZmDOF36* in association with starch synthesis, and characterized the regulation of starch synthesis gene expression in maize transgenic plants. Additionally, the molecular mechanism of ZmDOF36 action was clarified by a yeast one-hybrid assays and transient expression assays.

## Materials and Methods

### Plant Materials

Maize and rice seedlings were maintained at 28 ± 2°C under a 14 h light (3,500 lux)/10 h dark photoperiod at 70% humidity in a growth chamber. Roots, stems, leaves, tassels, *filaments, bracts*, embryos, and endosperm were sampled at 12, 14, 16, 18, and 20 days after pollination (DAP) from a single maize growth cycle were collected and immediately frozen in liquid nitrogen and stored at -80°C until use.

### Quantitative Real-Time PCR (qRT-PCR)

First-strand cDNA was synthesized from total plant RNA using the PrimeScript^TM^ RT reagent Kit with gDNA Eraser (Perfect Real Time; Takara). qRT-PCR assays were performed using SYBR Green qPCR Master Mix (Roche) in an ABI 7300 Real-Time System (Applied Biosystems). The PCR thermal cycling parameters were as follows: an initial 95°C denaturation for 10 min, followed by 40 cycles of 95°C for 15 s and 60°C for 1 min. The *ZmDOF36*-specific primer pair 5′-CCCGACAACTGATCCAATGC-3′ (forward) and 5′-GCATAACCACCGTCGTCGTT-3′ (reverse) were used in this study. The *ZmActin1* gene was used as an internal control for normalization of gene expression levels with the specific primers 5′-AGGCTTGTCTCCCAGGTCATC-3′ (forward) and 5′-GTTGGTCTGGAACTCGTTCACA-3′ (reverse). The sequences of the primers used to amplify the starch synthesis-related genes in the qRT-PCR assays are given in Supplementary Table S1.

### Gene Cloning and Production of Transgenic Plants

The coding region of the *ZmDOF36* gene (GRMZM2G137502_T02; 1,057 bp) from the maize B73 inbred line was amplified using the gene-specific primers 5′-CGGGATCCATGGACATGAACTCCAACGC-3′ (forward, *BamHI* site underlined) and 5′-CCGAATTCCTACCCCTCTGCCCCGTCGC-3′ (reverse, *EcoRI* site underlined). The PCR product was directly inserted into the plant over-expression vector pZZ00005 (China National Seed Group Co., Ltd.) for transformation of maize inbred line “ZZC01” (China National Seed Group Co., Ltd.) and the pCAMBIA1301 vector for transformation of rice (*Oryza sativa* L. subsp. *japonica* “Zhonghua 11”). Transgenic maize plants were identified using a protein test strip for bialaphos selection; 20 independent T_0_ transgenic lines were obtained, and the homozygous T_3_ transgenic lines were obtained for further analysis. *ZmDOF36* transgenic rice lines were selected by *GUS* staining and confirmed by PCR using specific primers to amplify the hygromycin resistance gene (HYG-F 5′-ACTCACCGCGACGTCTGT-3′ and HYG-R 5′-TTTCTTTGCCCTCGGACG-3′).

### Subcellular Localization of the ZmDOF36 Protein

The coding sequence of ZmDOF36 without the stop codon was amplified using specific primers 5′-GCTCTAGAATGGACATGAACTCCAACGC-3′ (forward, *XbaI* site underlined) and 5′-CGGGATCCCCCCTCTGCCCCGTCGCTGC-3′ (reverse, *BamHI* site underlined) and inserted into the pCAMBIA1305 vector under control of the cauliflower mosaic virus (CaMV) 35S promoter. The construct was transferred into *Agrobacterium tumefaciens* EHA105 cells, and a suspension of agrobacteria carrying the pCAMBIA1305-ZmDOF36 construct was infiltrated into emerging leaves of 40-day-old tobacco plants. GFP fluorescence signals were observed at 20× magnification using a Zeiss LSM 780 confocal laser scanning microscope after 2–3 days of incubation.

### Transcriptional Activation Analysis

The full-length (FL) or different truncated fragments of *ZmDOF36* were cloned separately into the pGBKT7 vector (Clontech) that carries a GAL4 DNA-binding domain. The sequences of PCR primers specific for these constructs are given in Supplementary Table S1. The Clontech yeast system was used in this study according to the manufacturer’s instructions. All constructs were transferred separately into cells of *Saccharomyces cerevisiae* strain AH109, and the interactions were detected on the amino acid-deficient media SD/Trp^-^ and SD/Trp^-^/His^-^/Ade^-^/X-α-gal at 30°C for 3–5 days.

### Determination of Agronomic Characters and Measurement of Starch Properties

Grain length, width, and thickness were measured using a vernier caliper from three randomly chosen, fully filled grains. The 1,000-grain weight was determined by weighing three replicates of 1000-grain samples independently on an electronic balance.

Endosperm was collected and then ground to a powder using a grinding mill. Finally, these powders are passed through a 50 mesh screen. The starch and amylose contents were measured using starch assay kits according to the manufacturer’s instructions (K-TSTA and K-AMYL; Megazyme). The Blue Value (BV) and maximum absorbance (λ_max_) of amylopectin were measured as described by Fu and Xue (2010). To determine the amount of soluble sugars, 50 mg of endosperm powder was washed twice in 80% (v/v) ethanol at 80°C for 40 min and assayed using anthrone reagent (Wang et al., 2013). To determine the amount of reducing sugars, 100 mg samples of powdered endosperm were boiled in a small amount of distilled water for 30 min in a 25 ml volumetric flask, cooled to room temperature, diluted to volume with distilled water, and assayed using the DNS colorimetric method (Miller, 1959).

### Scanning Electron Microscopy (SEM)

Seeds were dried completely under low pressure and cut across the short axis with a razor blade. The surfaces were then sputter coated with gold prior to observation with a Hitachi S-3000N SEM (Hitachi, Tokyo, Japan).

### Yeast One-Hybrid Assays

Promoter sequences containing the maize DOF elements (AAAG) from starch synthesis-related genes were inserted into the pAbAi vector to generate the bait vectors. The sequences of all primers used are given in Supplementary Table S1. The bait vectors were transformed into the Y1HGold yeast strain using the Yeastmaker^TM^ Yeast Transformation System 2 (Clontech, United States). The bait strains were screened on SD/-Ura medium plates containing 500 ng ml^-1^ Aureobasidin A (AbA). The prey vector carrying the pGADT7-ZmDOF36 construct was transformed into the bait strain and were screened on SD/-Leu-AbA^r^ defective plates for 3 days at 30°C.

### Transient Expression Assays in *Nicotiana benthamiana*

The full length *ZmDOF36* cDNA was cloned into the pCAMBIA1301 vector driven by the CaMV 35S promoter to generate the effector plasmid. The promoter regions of starch synthesis-related genes were separately cloned into the pBI121 vector, which was constructed by replacing the CaMV 35S promoter with the CaMV 35S minimal promoter, to generate the reporter plasmids (*Promoter*-35S mini-*GUS* gene cassettes). Suspensions of agrobacterium strain EHA105 cells carrying the effector plasmid were mixed in equal volumes with EHA105 cells carrying the reporter plasmids and infiltrated into *N. benthamiana* leaves. The *Agrobacterium*-mediated transient expression assays, GUS staining, and enzyme activity assays were performed as previously described (Yan et al., 2014; Wu et al., 2015).

### Statistical Analyses

All results were expressed as the mean values ± standard deviation (SD) of at least three replicates. Statistical analyses were performed using Microsoft Excel 2010 and SPSS v16.0 software (Chicago, IL, United States) based on Student’s *t*-test.

## Results

### *ZmDOF36* Is Predominantly Expressed in the Maize Endosperm

**FIGURE 1:**
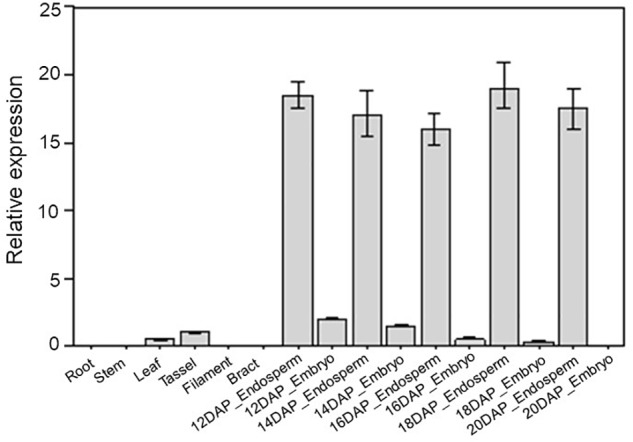
Tissue-specific expression patterns of *ZmDOF36* in maize by qRT-PCR. Relative expression was calculated by setting the expression of *ZmDOF36* in the tassel as 1.0. The *ZmActin1* gene was used as an internal control for normalization of gene expression. All bars represent means ± SD (*n* = 3 repeats). DAP, days after pollination.

Expression patterns can provide important clues for exploring gene function. Therefore, we collected maize tissues at 16 different developmental stages, including roots, stems, leaves, tassels, filaments, bracts, embryos, and endosperm at 12, 14, 16, 18, and 20 DAP. Total RNA was then extracted from these tissues to investigate the expression profile of *ZmDOF36* using qRT-PCR. The results of this experiment showed that *ZmDOF36* is mainly expressed in the endosperm (Figure 1), which is consistent with previously reported results (Li et al., 2014). Our results strongly suggest that ZmDOF36 may be involved in endosperm development.

### *ZmDOF36* Possesses the Typical Features of a Transcriptional Activator

**FIGURE 2:**
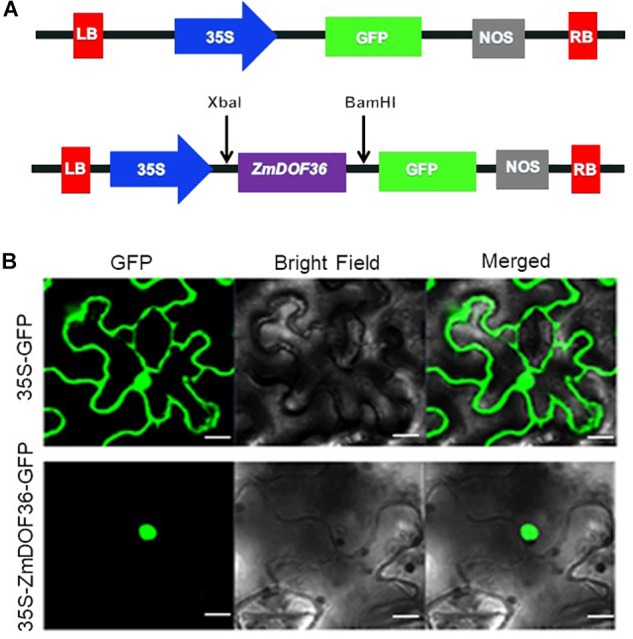
Subcellular localization of ZmDOF36. **(A)** Schematic diagram of the DNA construct used for ZmDOF36 subcellular localization. LB, T-DNA left border; 35S, cauliflower mosaic virus 35S promoter; GFP, green fluorescent protein; NOS, nopaline synthase gene terminator; RB, T-DNA right border. **(B)** The 35S::ZmDOF36-GFP fusion protein and 35S::GFP DNA constructs were transiently expressed separately in *Nicotiana benthamiana* leaf epidermal cells and visualized with a confocal laser scanning microscope. Scale bar = 20 μm.

To characterize the *ZmDOF36* gene, subcellular localization of the protein and transcriptional activity of the gene were investigated. We first generated the p1305-35S-*ZmDOF36-GFP* construct (Figure 2A) that was transiently expressed in *N. benthamiana* leaf epidermal cells. The ZmDOF36-GFP fusion protein fluorescence signal was detected almost exclusively in the nucleus by confocal laser-scanning microscopy, while the GFP control was detected both in the nucleus and cytoplasm (Figure 2B).

**FIGURE 3:**
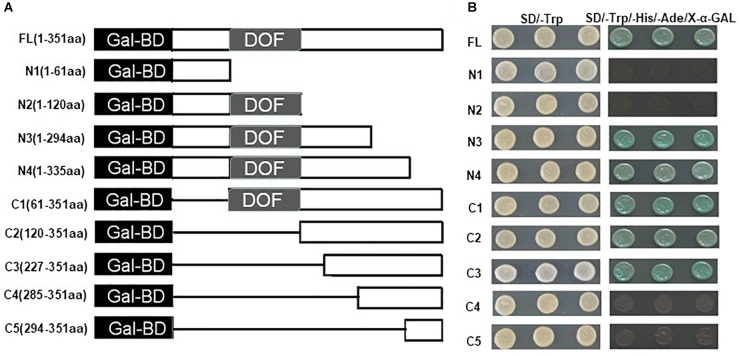
Transcription activation assay of full-length ZmDOF36 and nine different gene fragments in yeast. **(A)** Schematic representation of the *ZmDOF36* gene fragments used in the assays showing length and the relative positions of the deleted regions. Gal-BD, GAL4 DNA-binding domain. DOF, DNA binding Dof domain. **(B)** Transactivation activity analysis of the full-length *ZmDOF36* gene and the different gene fragments in yeast. The construct pGBKT7-ZmDOF36 was transformed into yeast strain AH109, containing *HIS3*, *ADE2*, and *MEL1* reporter genes. The results were determined by growth of the yeast strains SD/-Trp and SD/-Trp/-His/-Ade/X-α-GAL media for 3–5 days at 30°C.

A yeast assay system was then used to analyze the transcriptional activation activity of ZmDOF36. The full length *ZmDOF36* cDNA and several different truncated fragments were separately cloned into the pGBKT7 vector (Figure 3A). These constructs were then transformed into the yeast strain AH109. The results demonstrated that all transformed yeast strains grew well on SD/-Trp medium. The yeast strains carrying the positive control vector (pGBKT7-53 + pGADT7-T), the FL *ZmDOF36* (FL, 1-351 aa) and the N3, N4, C1, C2, and C3-terminus of ZmDOF36 grew well and appeared blue on the selective SD/Trp^-^/His^-^/Ade^-^/X-α-gal medium (Figure 3B), which shows that *ZmDOF36* is a transcriptional activator, and the active domain is mainly located in the C-terminus (amino acids 227–294) of the protein.

### *ZmDOF36* Affects the Seed Size in Transgenic Rice and Maize

**FIGURE 4:**
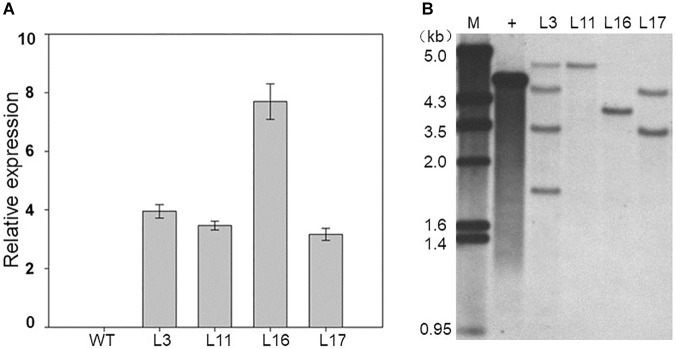
Molecular identification of *ZmDOF36*-expressing transgenic maize plants. **(A)** Expression levels of *ZmDOF36* in transgenic maize seedlings as determined by qRT-PCR. WT: wild-type (“ZZC01”). **(B)** Southern blot analysis of transgenic plants using the GUS gene as a probe. Genomic DNA was digested with EcoRI. M, λ/EcoRI DNA marker; +, plasmid DNA (pZZ00005-ZmDOF36) positive control. L3, L11, L16, and L17 are five independent transgenic lines that overexpress *ZmDOF36*.

**FIGURE 5:**
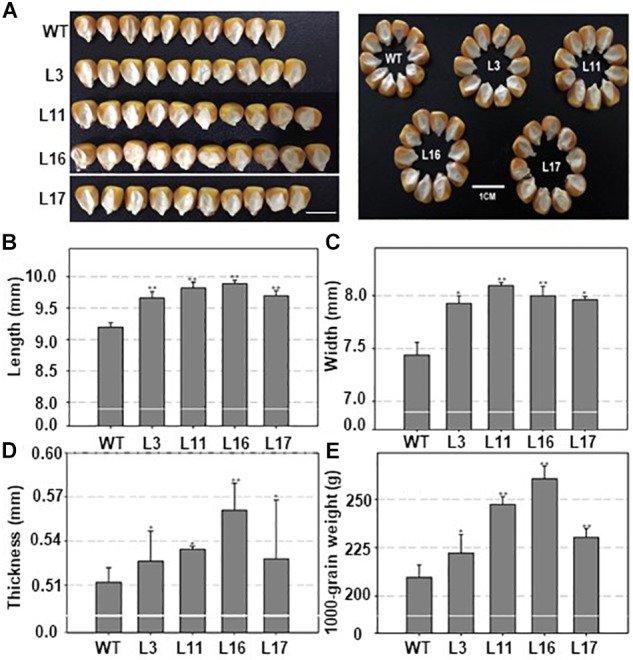
Seed morphology of wild type and *ZmDOF36*-overexpressing maize lines. **(A)** Seed morphology. **(B)** Seed length. **(C)** Seed width. **(D)** Seed thickness. **(E)** 1,000-grain weight of the seeds. WT, maize cultivar “ZZC01”; L3, L11, L16, and L17 are four independent transgenic maize lines overexpressing *ZmDOF36*. Data are presented as means ± SD of three replicates. ^∗^Significant differences between WT and the transgenic plants at *P* < 0.05. ^∗∗^Significant differences between WT and transgenic plants at < 0.01. Bar = 1 cm.

As mentioned above, *ZmDOF36* encodes a DOF-type TF that may be involved in endosperm development. To explore the function of ZmDOF36, we constructed the pCAMBIA1301-ZmDOF36 and pZZ00005-ZmDOF36 vectors in which expression of *ZmDOF36* is under control of the CaMV 35S promoter for rice and maize transformation, respectively. We generated 20 independent maize plants and 10 independent rice plants that over-expressed *ZmDOF36*. The expression levels and copy numbers of *ZmDOF36* in the transgenic plants were determined by qRT-PCR (Figure 4A and Supplementary Figure S1) and Southern blot analysis (Figure 4B and Supplementary Figure S2), respectively. Rice and maize are two closely related crop species in the grass family. The results of studies in transgenic rice can support the functions of genes in transgenic maize. So four T_3_-generation maize transgenic lines and three T_3_-generation rice transgenic lines were used for further experiments. Seed length, width, thickness, and the 1000-grain weights were measured in the wild-type (WT) and *ZmDOF36*-overexpressing lines. The results showed that all parameters were apparently increased in the transgenic maize lines compared with WT, especially in the single-copy lines L11 and L16 (Figure 5). Similarly, we also measured the seed parameters in the *ZmDOF36*-overexpressing rice lines and WT. Compared with the WT, the grain lengths and 1000-grain weights were increased in the transgenic lines (Supplementary Figures S3A,B,D), while there was no difference in grain thickness between the transgenic and WT rice lines (Supplementary Figure S3C).

### *ZmDOF36* Affects the Starch Content and Structure in Transgenic Maize Endosperm

**FIGURE 6:**
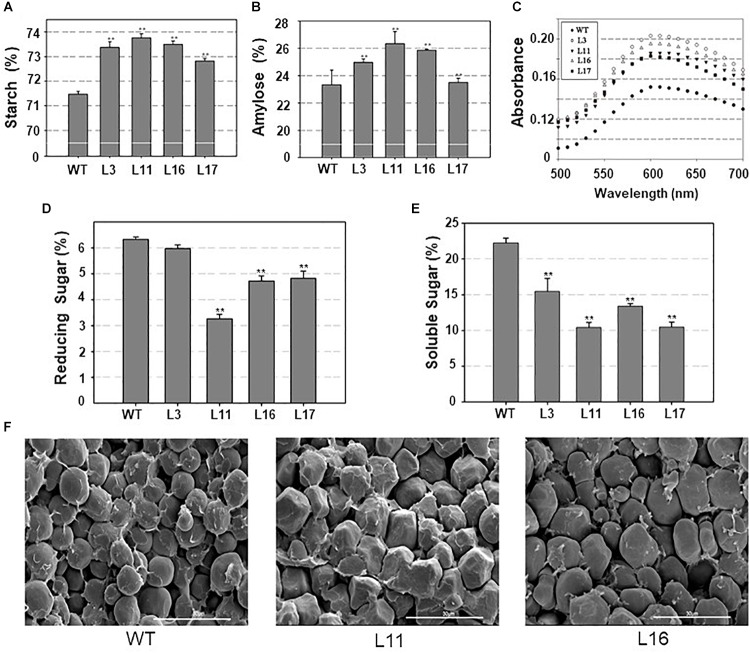
Altered starch content and structure in the *ZmDOF36*- overexpressing maize lines **(A)** Total starch content. **(B)** Amylose content. **(C)** Absorbance spectra of isolated amylopectin-iodine complexes. Amylopectin was separated from total starch extracts and then reacted with iodine solution. BV at 680 nm and λ_max_ indicate the ability of starch to combine with iodine. **(D)** Reducing sugar content. **(E)** Soluble sugar content. **(F)** Scanning electron microscopy (SEM) images of transverse sections of maize seeds. ^∗∗^Significant differences between WT and transgenic plants at *P* < 0.01. Scale bar = 30 μm.

To understand the effects of *ZmDOF36* on starch synthesis, we analyzed the total starch and amylose contents in maize and rice seeds. The results showed that the average total starch and amylose contents of the *ZmDOF36* over-expressing maize lines were higher than in the WT (Figure 6A,B). In rice, the total starch and amylose contents showed similar trends (Supplementary Figure S4). We assayed the soluble sugar and reducing sugar contents because they have been reported to be involved in starch accumulation (Kashem et al., 1995). The results showed that seeds of maize lines over-expressing *ZmDOF36* had significantly lower levels of soluble sugars and reducing sugars compared with WT (Figure 6D,E).

The BV and maximum absorption wavelength (λ_max_) can provide important clues about starch structure (Hizukuri, 1986; Takeda et al., 1986). To determine whether the amylopectin structure had been altered by overexpression of *ZmDOF36*, we measured the BV and λ_max_ of amylopectin extracted from seeds of the transgenic lines. The results revealed that for the amylopectin, both BV and λ_max_ were slightly increased in the transgenic maize lines compared to the WT (Figure 6C), suggesting that *ZmDOF36* over-expression results in a higher fraction of long branched starch chains in maize seeds.

In order to further investigate the influence of *ZmDOF36* on starch structure, we used SEM to observe transverse seed sections. The results showed that the WT maize endosperm is filled with smooth, spherical starch granules, while the endosperm of *ZmDOF36-*expressing transgenic lines had a looser, irregular polyhedron structure packed with larger starch granules (Figure 6F).

### *ZmDOF36* Influences the Expression Profiles of Numerous Starch Synthesis-Related Genes

**FIGURE 7:**
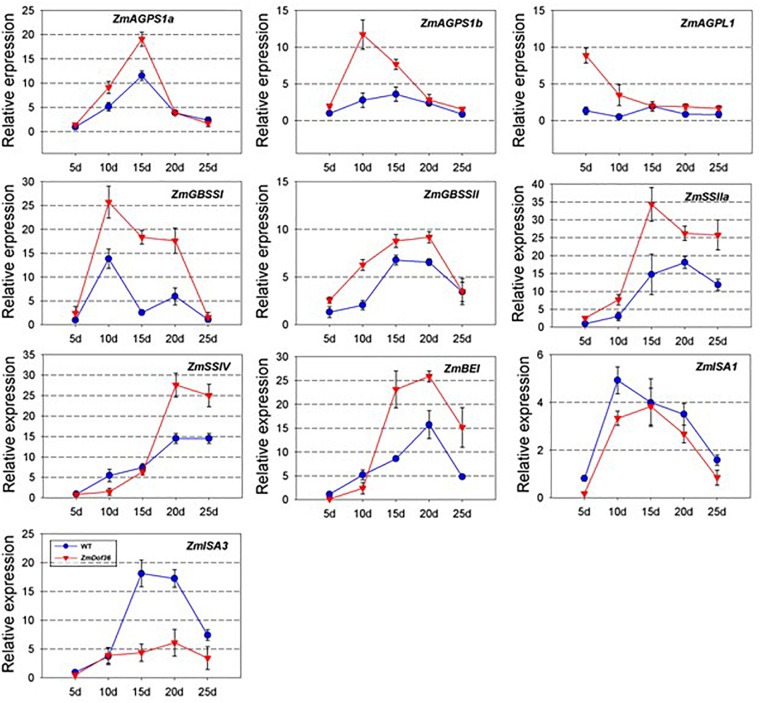
Relative expression levels of 10 maize starch synthesis genes during seed development in the wild-type and *ZmDOF36-*overexpressing lines. The mixed sample of four genetically modified maize lines was used to analyze the gene expression. The expression of each gene in 5 DAF endosperm of maize line “ZZC01” was used as the control. Values represent the mean ± SD of three replicates.

ADP glucose pyrophosphorylase (AGPase), GBSS, soluble SS, starch BE, and starch DBE are key enzymes in the starch biosynthesis pathway in the maize endosperm (Whitt et al., 2002; Jeon et al., 2010). To determine the regulatory role of ZmDOF36 in the expression of starch synthesis genes, qRT-PCR was performed to analyze the expression of 10 starch biosynthesis genes in transgenic maize endosperm sampled at 12–20 DAP. Compared with the WT, the expression of most starch synthesis genes including *ZmAGPS1a*, *ZmAGPS1b, ZmAGPL1*, *ZmGBSS1*, *ZmGBSSIIa*, *ZmSSIIa*, *ZmSSIV*, and *ZmBE1* was increased in the *ZmDOF36*-overexpressing maize lines throughout seed development, especially that of *ZmGBSSI*, which was upregulated by ∼6-fold compared to the wild type at 15 DAP, although the relative extent of the increase varied for the different genes and developmental stages, whereas *ZmISA1* and *ZmISA3* were down-regulated in the transgenic lines compared with the WT over the course of the experiment (Figure 7). Starch synthesis peaks at 12–15 DAP and continues throughout later kernel development in maize endosperm (Scanlon and Takacs, 2009), which is consistent with the expression trends observed for the starch synthesis genes. Interestingly, *ZmDOF36* was highly expressed in 12–15 DAP endosperm in both our results (Figure 1) and previous study (Li et al., 2014). This result demonstrated that *ZmDOF36* might participate in the transcriptional control of the starch synthesis-related genes.

### *ZmDOF36* Activates the Expression of ZmGBSSI and ZmISA1 by Binding to the Promoters in Yeast and Plant Cells

**FIGURE 8:**
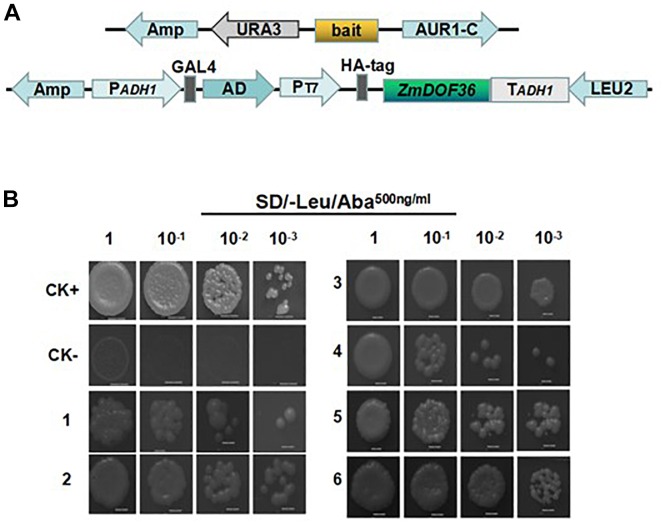
Analysis of the interaction between ZmDOF36 and the promoter regions of six starch synthesis genes in yeast one-hybrid assays. **(A)** Schematic diagrams of the bait and reporter constructs. **(B)** Screening on SD/-Leu medium containing 500 ng/mL Aureobasidin A (AbA); **CK**+, p53AbAi-p53 fragment+pGADT7-Rec (SmaI-linearized); **CK-**, p53AbAi+pGADT7-ZmDOF36; **1,** *pZmAGPS1a*; **2,** *pZmAGPL1*; **3,** *pZmGBSSI*; **4,** *pZmSSIIa*; **5,** *pZmISA1*; **6.** *pZmISA3.*

Previous studies have shown that DOF proteins can bind directly to AAAG sequence motifs (Yanagisawa and Schmidt, 1999). Analysis of promoter sequences using the online software PlantCARE showed that the specific motif sequence (AAAG) can be identified in the promoters of the starch synthesis-related genes (data not shown). To further explore the functional mechanism of ZmDOF36 activity in maize starch synthesis, we used the yeast one-hybrid assay to investigate whether ZmDOF36 can bind to the promoters of the starch synthesis-related genes. The results showed that the yeast cells co-expressing *ZmDOF36* and *pZmAGPS1a, pZmAGPL1, pZmGBSSI*, *pZmSSIIa*, *pZmISA1*, or *pZmISA3* (Figure 8A) grew normally on the selection medium (Figure 8B), but yeast cells co-expressing *ZmDOF36* and *pZmAGPS1b*, *pZmGBSSIIa*, *pZmSSIV*, or *pZmBE1* could not grow under selection (Supplementary Figure S5). These results suggest that ZmDOF36 can bind to the *pZmAGPS1a, pZmAGPL1, pZmGBSSI*, *pZmSSIIa*, *pZmISA1*, and *pZmISA3* gene promoters directly in yeast cells. In addition, the results of our yeast one-hybrid analysis explain why the expression levels of starch synthesis genes, particularly GBSSI1, SSII, and ISA1, were strongly influenced in our transient expression experiments.

**FIGURE 9:**
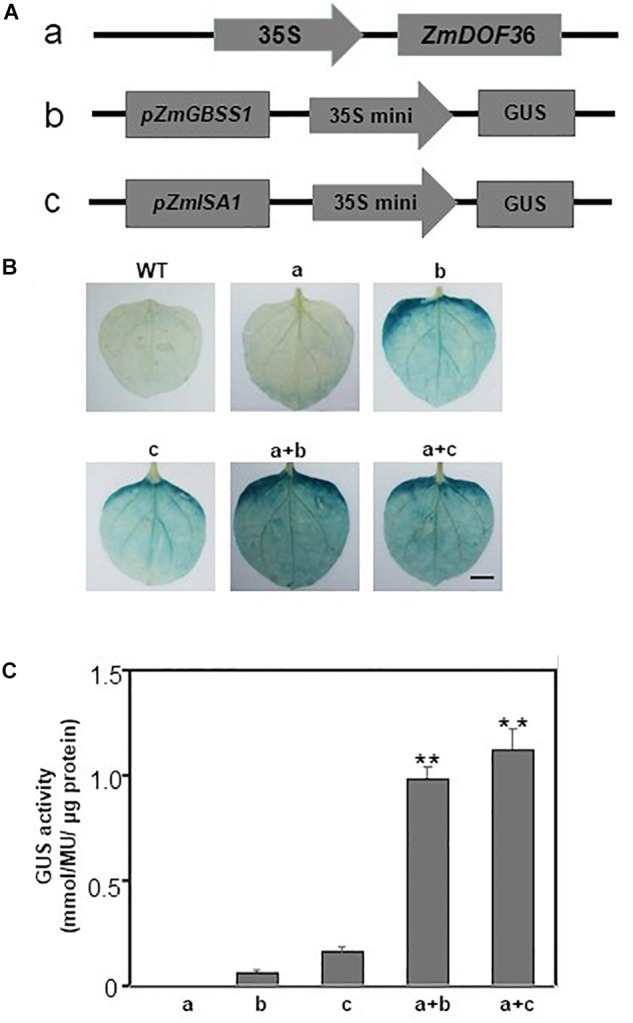
Transactivation activity of ZmDOF36 in *N. benthamiana* leaves **(A)** Schematic diagrams of the effector and reporter constructs. **(B)** Histochemical analysis of GUS expression in *N. benthamiana* leaves. Fully expanded leaves from 8-week-old seedlings were agroinfiltrated with the indicated reporter and effector plasmids at an OD_600_ of 0.6. **(C)** GUS fluorescence quantitative analysis of *N. benthamiana* leaves from **(B)**. Values are reported as means ± SD of three replicate analyses. ^∗∗^Significant differences between WT and transgenic plants at *P* < 0.01. Scale bar = 10 mm.

To confirm whether ZmDOF36 activates the expression of *pZmAGPS1a, pZmAGPL1*, *pZmGBSSI*, *pZmSSIIa*, *pZmISA1*, and *pZmISA3* in a plant system, we performed transient co-expression assays in *N. benthamiana* leaves. The different reporter vector *promoter*-35S mini-GUS constructs (Figure 9A) were infiltrated either alone or with the effector plasmid 35S:ZmDOF36 construct (Figure 9B) into *N. benthamiana* leaves followed by GUS histochemical staining and a quantitative GUS fluorescence assay. Leaves infiltrated with the effector plasmid showed a light blue background when stained for GUS activity and a low level of expression of the GUS gene, and uninfiltrated leaves showed no GUS activity. Leaves infiltrated individually with either of the reporter vectors showed light blue staining and a background level of GUS enzymatic activity. In contrast, leaves co-transformed separately with *pZmGBSSI*-35S mini-GUS or *pZmISA1*-35S and *35S:ZmDOF36* showed dark GUS staining and significantly increased levels of GUS enzyme activity (Figure 9B,C). However, leaves co-transformed with *pZmAGPS1a*, *pZmAGPL1*, *pZmSSIIa*, and *pZmISA3* and *35S:ZmDOF36* exhibited no obvious difference in GUS expression levels compared with that the reporter vector alone (data not shown).

## Discussion

The DOF TF family is a class of plant-specific TFs that are widely involved in plant growth, development, signal transduction, and the response to biotic and abiotic stress (Yanagisawa, 2002; Noguero et al., 2013). Although the DOF proteins PBF and DOF1, both of which have been reported to be involved in seed development and carbon metabolism (Yanagisawa, 2000; Hwang et al., 2004; Marzabal et al., 2008), direct evidence for DOF protein regulation of starch biosynthesis in maize is not well established. In the present study, we report the isolation and characterization of a maize DOF protein, ZmDOF36, and show that it regulates starch synthesis in transgenic maize.

We found that ZmDOF36 is localized to the nucleus, possesses transcriptional activation activity, and is able to bind to promoter regions of starch synthesis genes containing the AAAG motif, results which are consistent with the features of DOF TFs (Yanagisawa and Schmidt, 1999). In addition, analysis of the gene expression profile showed that *ZmDOF36* is predominantly expressed in the maize endosperm. Together, these results suggest that *ZmDOF36* is a typical DOF-type transcriptional activator and may be involved in regulating the process of starch synthesis in maize.

Starch is the primary carbohydrate in the diets of humans and livestock, and has a tremendous economic value. Starch synthesis is a complex biological process that is catalyzed by a large number of functional enzymes, such as AGPase, SS, GBSS, BE, and DBE (Jeon et al., 2010). Starch biosynthesis cannot be manipulated in heterologous systems or *in vitro*. Therefore, transgenic maize lines overexpressing *ZmDOF36* were generated to investigate the starch metabolic processes. Starch synthesis peaks at 12–15 DAP in the maize endosperm, and continues throughout later phases of kernel development (Scanlon and Takacs, 2009). Compared to the WT, we measured increased expression of *ZmAGPS1a* (*bt2*), *ZmAGPS1b, ZmAGPL1* (*sh2*), and *ZmGBSS1* at 10–15 DAP in seeds of the transgenic lines. Importantly, *ZmDOF36* was also highly expressed in 12–15 DAP endosperm in both our results and previous study (Li et al., 2014). This result demonstrated that *ZmDOF36* might participate in the transcriptional control of the starch synthesis-related genes. Furthermore, yeast one-hybrid assays showed that ZmDOF36 is able to bind to the promoters of *pZmAGPS1a*, *pZmAGPL1*, and *pZmGBSSI*. AGPase is a rate-limiting enzyme that catalyzes the first unique step in starch synthesis, and the enzyme consists of two large subunits (AGPase LS) and two small subunits (AGPase SS) (Huang et al., 2014). Over-expression of AGPase genes can enhance starch content in transgenic maize (Li et al., 2011). The *waxy* (*wx*) gene encodes GBSSI, and mutations at this locus result in the production of starch granules that lack amylose and contain only amylopectin (Macdonald and Preiss, 1985). This may explain in part why we measured high starch and amylose contents in *ZmDOF36*-expressing transgenic seeds. In addition, compared with WT, over-expression of the *ZmDOF36* gene resulted in reduced levels of soluble sugars and reducing sugars in the endosperm of the seeds from transgenic plants. This indicated that over-expression of the *ZmDOF36* gene directed more soluble sugar toward starch synthesis.

In addition to starch content, we also analyzed starch structure. Many studies have shown that BV and λ_max_ are two major indexes that indicate the capacity of starch to combine with iodine. Different BV and k_max_ values reflect differences in starch structure (Takeda et al., 1986; Hizukuri, 1988). Compared with the WT, BV, and λ_max_ as determined for amylopectin were slightly increased in the transgenic maize seeds. This result is also further demonstrated by the SEM result, which showed a loose, irregular polyhedron structure packed with larger starch granules in the endosperm of *ZmDOF36* transgenic lines. SSs, Bes, and DBEs cooperatively determine the fine structure of amylopectin in a highly complex manner (Ohdan et al., 2005). Our results also show that the expression of *ZmGBSSIIa*, *ZmSSIIa*, *ZmSSIV*, and *ZmBE1* was up-regulated, while *ZmISA1* and *ZmISA3* were down-regulated in the transgenic lines compared with WT. Changes in the relative expression levels of these genes can partially account for the observed change in starch granule size in the transgenic lines. Additional experiments will be needed to characterize the precise effect that *ZmDOF36* over-expression has on the mechanism of starch synthesis, because the results of yeast one-hybrid and transient co-expression assays did not show that ZmDOF36 can bind the promoter and increase the expression of all starch synthesis genes.

Interestingly, transgenic maize plants also had increased seed size and weight compared with the WT. In fact, previous studies have also suggested that rice productivity and yield are primarily sink-limited in source-sink relationships during seed development (Okita et al., 2001). Thus, overexpression of ZmDOF36 may result in enhanced starch accumulation, which in turn leads to an improvement in the capacity to utilize the initial photosynthetic product, resulting in increased weight and yield. Over-expression of AGPase genes has been shown to increase seed size in transgenic maize (Li et al., 2011). We found that AGPase genes were indeed up-regulated in *ZmDOF36* transgenic maize plants. Therefore, our results provide a potential strategy for breeding new maize inbred lines with larger seeds and higher starch content. Obviously, further research will be needed in the future.

In conclusion, the results of our study demonstrate that ZmDOF36 is a candidate regulator of starch biosynthesis in maize. Overexpression of *ZmDOF36* in maize increases both seed starch content and seed size. In addition, ZmDOF36 can positively regulate the expression of starch synthesis genes by binding to a specific sequence motif in the promoter regions. Taken together, our data suggests that ZmDOF36 acts as an important regulatory factor of starch synthesis by binding to the promoters of starch synthesis genes to regulate their expression in maize endosperm.

## Author Contributions

JW, LC, and BC conceived the experiments. JW, LC, MC, WZ, and QD performed the research and collected the data. JW, LC, HJ, and BC analyzed the data and wrote the manuscript. All authors reviewed and approved this submission.

## Conflict of Interest Statement

The authors declare that the research was conducted in the absence of any commercial or financial relationships that could be construed as a potential conflict of interest.

## Supplementary Material

FIGURE S1 Expression of *ZmDOF36* in transgenic rice lines. **(A)**, Relative expression levels of *ZmDOF36* in transgenic rice seedlings as determined by RT-PCR. WT: Wild type (“Zhonghua 11”). L2, L3, L5: three different transgenic rice lines overexpressing *ZmDOF36*. **(B)** Expression levels of WT and three selected *ZmDOF36* transgenic lines as determined using qRT-PCR. *Actin1* was used as an RNA loading standard for comparison to *ZmDOF36* expression levels.

FIGURE S2 Southern blot analysis of *ZmDOF36*-expressing transgenic rice lines. Genomic DNA was digested with EcoRI. M, DNA Molecular-Weight Marker III; +, Plasmid control (pCAMBIA1301-ZmDOF36). L2, L3, and L5 are three independent transgenic lines overexpressing *ZmDOF36*.

FIGURE S3 Seed morphology of wild type and *ZmDOF36*-overexpressing rice lines. **(A)** Seed morphology. **(B)** Seed length. **(C)** Seed thickness. **(D)** 1,000-grain seed weights. WT, maize cultivar “Zhonghua 11.” L2, L3, and L5 are three independent transgenic rice lines expressing *ZmDOF36*. Data are presented as means ± SD from three replicates. ^∗∗^Significant differences between WT and transgenic plants at *P* < 0.01.

FIGURE S4 Altered starch content in seeds of *ZmDOF36-*overexpressing rice lines. **(A)** Total starch content. **(B)** Amylose content. ^∗^Significant differences between WT and the transgenic plants (L2, L3, and L5) at *P* < 0.05. ^∗∗^Significant differences between WT and the transgenic plants (L2, L3, and L5) at *P* < 0.01.

FIGURE S5 Analysis of the interactions between ZmDOF36 and the promoters of four starch synthesis genes as determined in yeast one-hybrid assays. **CK**+, p53AbAi-p53 fragment+pGADT7-Rec (SmaI-linearized); **CK**-, p53AbAi+pGADT7-ZmDOF36; **1,** *pZmAGPS1b*; **2,** *pZmGBSSII*; **3,** *pZmSSIV*; **4,** *pZmBE1.* SD/-Leu/Aba^500ng/ml^ is SD minimal medium without leucine, supplemented with 500 ng ml^-1^ Aureobasidin A.

TABLE S1 Oligonucleotide primers used in this study.
