# Supplementary figures and images for: The DOF-Domain Transcription Factor ZmDOF36 Positively Regulates Starch Synthesis in Transgenic Maize
Source: Front Plant Sci. 2019 Apr 12;10:465. doi: 10.3389/fpls.2019.00465 (PMC6474321; doi:10.3389/fpls.2019.00465)

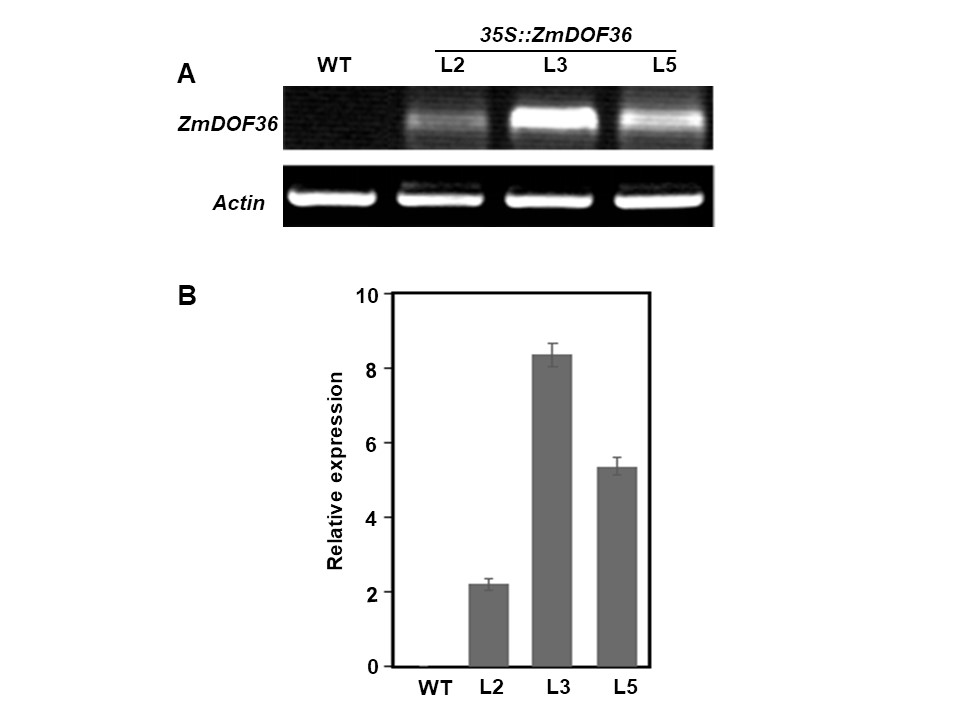

Supplement: FIGURE S1 — Expression of ZmDOF36 in transgenic rice lines. (A), Relative expression levels of ZmDOF36 in transgenic rice seedlings as determined by RT-PCR. WT: Wild type (“Zhonghua 11”). L2, L3, L5: three different transgenic rice lines overexpressing ZmDOF36. (B) Expression levels of WT and three selected ZmDOF36 transgenic lines as determined using qRT-PCR. Actin1 was used as an RNA loading standard for comparison to ZmDOF36 expression levels. [file Image_1.JPEG]

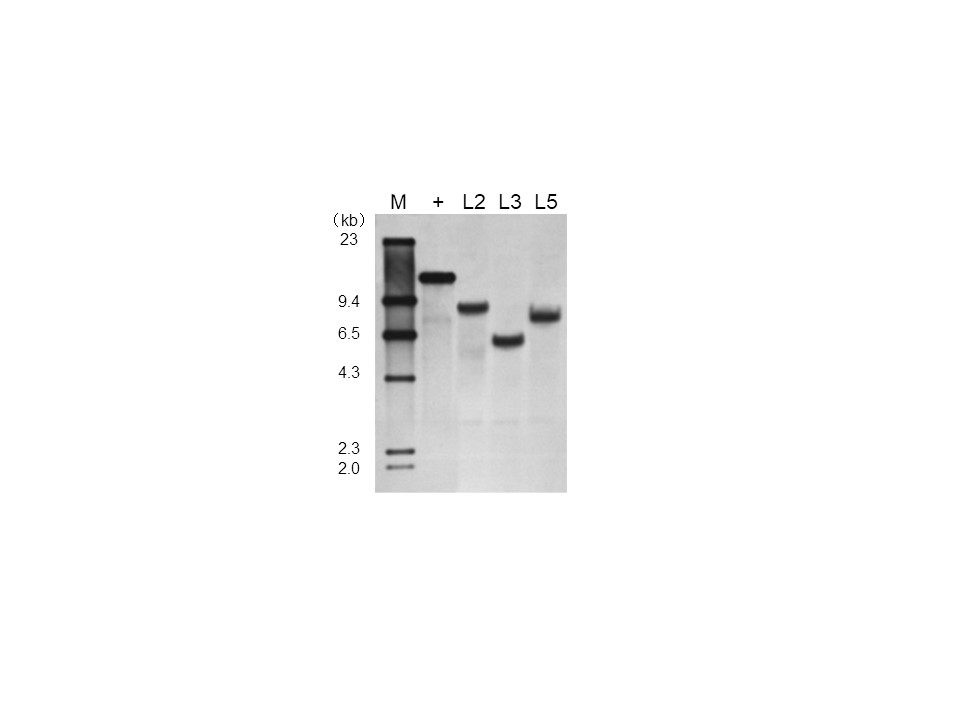

Supplement: FIGURE S2 — Southern blot analysis of ZmDOF36-expressing transgenic rice lines. Genomic DNA was digested with EcoRI. M, DNA Molecular-Weight Marker III; +, Plasmid control (pCAMBIA1301-ZmDOF36). L2, L3, and L5 are three independent transgenic lines overexpressing ZmDOF36. [file Image_2.JPEG]

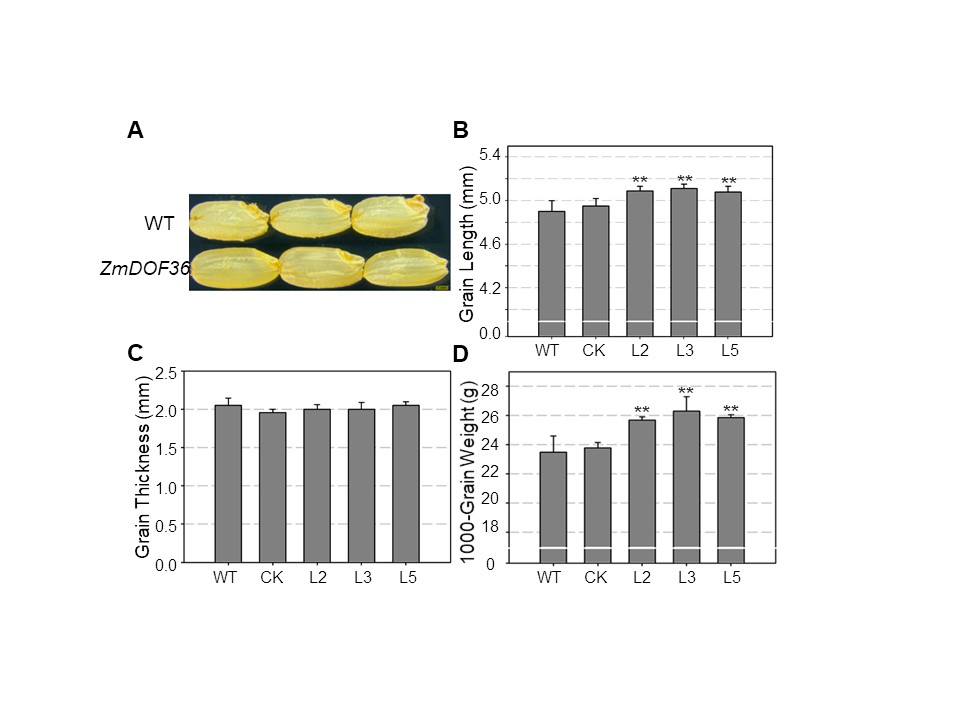

Supplement: FIGURE S3 — Seed morphology of wild type and ZmDOF36-overexpressing rice lines. (A) Seed morphology. (B) Seed length. (C) Seed thickness. (D) 1,000-grain seed weights. WT, maize cultivar “Zhonghua 11.” L2, L3, and L5 are three independent transgenic rice lines expressing ZmDOF36. Data are presented as means ± SD from three replicates. ∗∗Significant differences between WT and transgenic plants at P < 0.01. [file Image_3.JPEG]

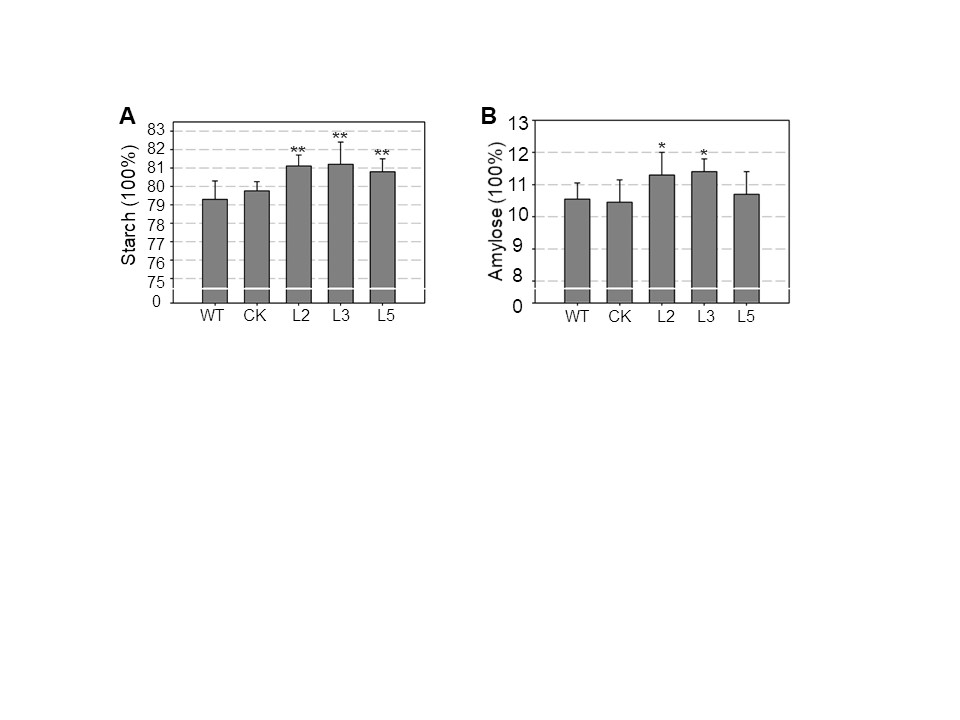

Supplement: FIGURE S4 — Altered starch content in seeds of ZmDOF36-overexpressing rice lines. (A) Total starch content. (B) Amylose content. ∗Significant differences between WT and the transgenic plants (L2, L3, and L5) at P < 0.05. ∗∗Significant differences between WT and the transgenic plants (L2, L3, and L5) at P < 0.01. [file Image_4.JPEG]

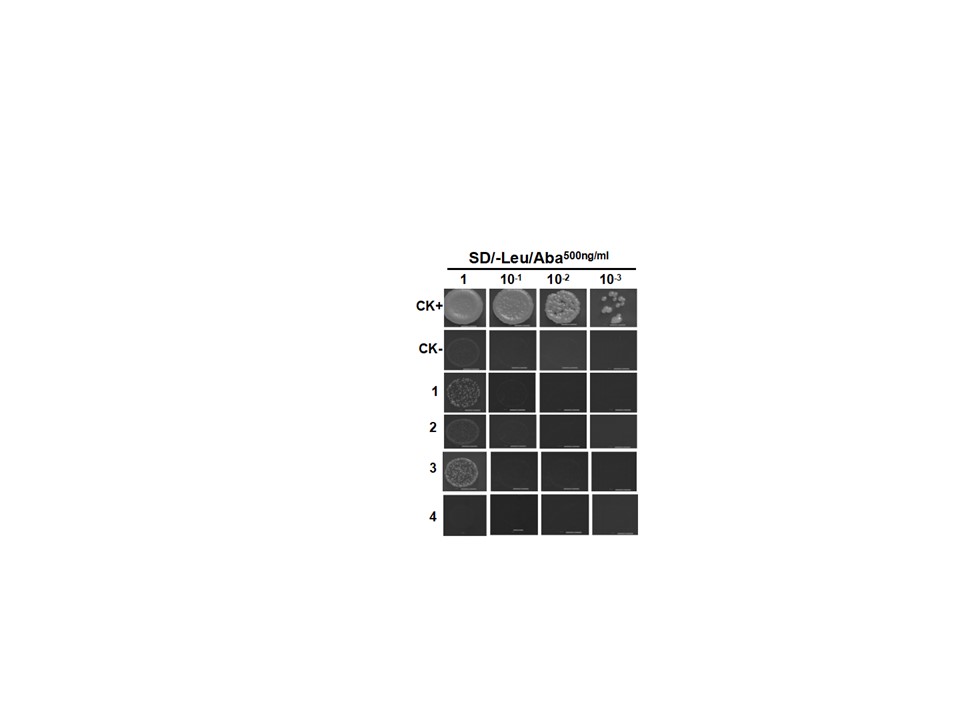

Supplement: FIGURE S5 — Analysis of the interactions between ZmDOF36 and the promoters of four starch synthesis genes as determined in yeast one-hybrid assays. CK+, p53AbAi-p53 fragment+pGADT7-Rec (SmaI-linearized); CK-, p53AbAi+pGADT7-ZmDOF36; 1, pZmAGPS1b; 2, pZmGBSSII; 3, pZmSSIV; 4, pZmBE1. SD/-Leu/Aba500ng/ml is SD minimal medium without leucine, supplemented with 500 ng ml-1 Aureobasidin A. [file Image_5.JPEG]
