# Supplementary material for: The DOF-Domain Transcription Factor ZmDOF36 Positively Regulates Starch Synthesis in Transgenic Maize
Source: Front Plant Sci. 2019 Apr 12;10:465. doi: 10.3389/fpls.2019.00465 (PMC6474321; doi:10.3389/fpls.2019.00465)
Supplement: TABLE S1 — Oligonucleotide primers used in this study. [file Table_1.DOC]

**TABLE S1 Primers used in this work.**

| **Assay** | **Primer Name** | **Sequence (5'-3')** |
| --- | --- | --- |
| **qRT-PCR analysis** | ZmAGPS1a-1-F | GCACAGCAGAGCCCAGATAA |
|  | ZmAGPS1a-1-R | CCATCCGGTACAGGTGATCG |
|  | ZmAGPS1b-F | AGCCTACGGGAACAACATTGG |
|  | ZmAGPS1b-R | CCCTGAAACCAGTTTGGATTATCT |
|  | ZmAGPL1-F | AGCCACAAGATCACTTCGGG |
|  | ZmAGPL1-R | GGTGAGGACCTGAGTTCGTG |
|  | ZmGBSSI-F | TGACGCTGGAACGGACTAC |
|  | ZmGBSSI-R | TCAAGTGCTGCCTGGCATAG |
|  | ZmGBSSIIa-F | ACCGGATTCCACATGGGTTC |
|  | ZmGBSSIIa-R | GAAGCACCTCCTCCCACTTC |
|  | ZmSSIIa-F | TTGATGCCCCTCTTTTCCGG |
|  | ZmSSIIa-R | TCAGGCAAGTCCATGTACGG |
|  | ZmSSIV-F | GGATAATCGCCTCCGGGAAG |
|  | ZmSSIV-R | GAAGTGCCTTCCCAAGACCA |
|  | ZmBEI-F | AATGGCCCTTGGAGGTGATG |
|  | ZmBEI-R | CGCTTGGTCAAACGCATTCA |
|  | ZmISA1-F | TGCCCTGCCATGAGTTCAAT |
|  | ZmISA1-R | GGGCCTCCCTTACAAAAGCT |
|  | ZmISA3-F | ATGGCATGTGTCAGTGGAGG |
|  | ZmISA3-R | GTTCCGAAAAGCTGGCTTGG |
|  | ZmTub-F | TGAGGGAGTGCATCTCGATC |
|  | ZmTub-R | CCTCCCCCAATGGTCTTGTC |
| **Transcriptional activation assay** | FL-F | CGGAATTCATGGACATGAACTCCAACGC |
|  | FL-R | CGGGATCCCTACCCCTCTGCCCCGTCGC |
|  | N1-R | CGGGATCC CGGCGGAGGCCGCGGCCTAG |
|  | N2-R | CGGGATCC CAGGAAAGACGGCGTCGTGT |

|  | N3-R | CGGGATCC CTACCCCTCTGCCCCGTCGC |
| --- | --- | --- |
|  | N4-R | CGGAATTC TCCAGCTCCTCGTCCCCG |
|  | C1-F | CGGAATTC GGTTATGCAGCAGGACCAGC |
|  | C2-F | CGGAATTCGTGCAGCAGCAGCAGCAGCA |
|  | C3-F | CGGAATTC GAGAACAAAGAGGCGGGGAA |
|  | C4-F | CGGAATTC ATGGACATGAACTCCAACGCC |
|  | C5-F | CGGAATTC ATGGACATGAACTCCAACGCCA |
|  | C6-F | CGGAATTC GACGCGGCGCACAGCTGC |
| **Yeast one hybrid analysis** | pZmAGPS1a-1-F | CCCAAGCTTATCGTGCATGGATGTATAATGACTG |
|  | pZmAGPS1a-1-R | GGGCTCGAGTCGGTTAATGACAAGGAGAATACGT |
|  | pZmAGPS1b-F | CCAAGCTTTGACCTGACTTACTCCACCT |
|  | pZmAGPS1b-R | CCCTCGAGATGCTTTCAAATTGGTCACT |
|  | pZmAGPL1-F | CCCAAGCTTTGGTGCTTCTGTTAGATGTTGG |
|  | pZmAGPL1-R | GGGCTCGAGAACGTATCCGGTGTTCAAAGGGGT |
|  | pZmGBSSI-F | CCCAAGCTTTACGTACGACGAAGACACGGAAGCC |
|  | pZmGBSSI-R | GGGCTCGAGAGACGAGGAGTACCAGCACAGCACG |
|  | pZmGBSSIIa-F | CCCAAGCTTGATCCGGACACACGTTAAAGCGAAG |
|  | pZmGBSSIIa-R | GGGCTCGAGCTGGACGTGTCACCTTGGCTTGGGG |
|  | pZmSSIIa-F | CCCAAGCTTGCTCCTCCTACCACGTCCACGA |
|  | pZmSSIIa-R | GGGCTCGAGTGAGCGGGTGTCGTCCTCGGAG |
|  | pZmSSIV-F | CCAAGCTTTGTGGGCTGTGTAAAACCTG |
|  | pZmSSIV-R | CCCTCGAGCCGACGGGTTCAAAAATTTA |
|  | pZmBEI-F | CCCAAGCTTACTCGCTTCCCGTGGGCTGC |
|  | pZmBEI-R | GGGCTCGAGCGCCCCACCCAGCAGCAGAT |
|  | pZmISA1-F | CCCAAGCTTCCCGTTCACGTTTCGCAGCTTTCGC |
|  | pZmISA1-R | GGGCTCGAGGATGAGTAGGGGAGTTATTAAGTCT |
|  | pZmISA3-F | CCCAAGCTTACAATGTTCAGTTATGTAATCAC |
|  | pZmISA3-R | GGGCTCGAGTTAGATGGCAAACCGAATG |
